# Vocal development through morphological computation

**DOI:** 10.1371/journal.pbio.2003933

**Published:** 2018-02-20

**Authors:** Yisi S. Zhang, Asif A. Ghazanfar

**Affiliations:** 1 Princeton Neuroscience Institute, Princeton University, Princeton, New Jersey, United States of America; 2 Department of Psychology, Princeton University, Princeton, New Jersey, United States of America; 3 Department of Ecology & Evolutionary Biology, Princeton University, Princeton, New Jersey, United States of America; Emory University, United States of America

## Abstract

The vocal behavior of infants changes dramatically during early life. Whether or not such a change results from the growth of the body during development—as opposed to solely neural changes—has rarely been investigated. In this study of vocal development in marmoset monkeys, we tested the putative causal relationship between bodily growth and vocal development. During the first two months of life, the spontaneous vocalizations of marmosets undergo (1) a gradual disappearance of context-inappropriate call types and (2) an elongation in the duration of context-appropriate contact calls. We hypothesized that both changes are the natural consequences of lung growth and do not require any changes at the neural level. To test this idea, we first present a central pattern generator model of marmoset vocal production to demonstrate that lung growth can affect the temporal and oscillatory dynamics of neural circuits via sensory feedback from the lungs. Lung growth qualitatively shifted vocal behavior in the direction observed in real marmoset monkey vocal development. We then empirically tested this hypothesis by placing the marmoset infants in a helium–oxygen (heliox) environment in which air is much lighter. This simulated a reversal in development by decreasing the effort required to respire, thus increasing the respiration rate (as though the lungs were smaller). The heliox manipulation increased the proportions of inappropriate call types and decreased the duration of contact calls, consistent with a brief reversal of vocal development. These results suggest that bodily growth alone can play a major role in shaping the development of vocal behavior.

## Author summary

In robotics, the shape and material properties of the robot body can be exploited to make central control processes simpler; this is known as “morphological computation.” In this view, the body is not a device to simply be controlled by the brain, but rather is directly involved in making some behaviors less complicated for the nervous system. We tested this idea in a real biological system by investigating how marmoset monkey infants change their vocal behavior over time. It would typically (and reasonably) be presumed that changes in vocal production are the result of learning and, thus, changes in the nervous system. However, using a computational model, we show that one major feature of developing vocal behavior—the decline in the production of context-inappropriate vocalizations—could simply be the result of lung growth (a change in body morphology) without any concomitant changes in central nervous system structure. We then tested the model predictions by placing the infants in a helium–oxygen (heliox) environment. This, in effect, simulated a reversal in lung growth and, as predicted, resulted in a reversion back to immature vocal behavior. Thus, morphological computation plays a role in vocal development. These data underscore the importance of considering the whole organism, not just the nervous system, when trying to understand how any behavior works or may go awry.

## Introduction

It is well established (though often ignored) that central pattern generators (CPGs) are constrained and modulated by the body in which they are embedded [1,2]. Moreover, in the field of robotics, much work has demonstrated how the shape and material properties of the body can be exploited to make analogous central control processes simpler; this is known as “morphological computation” [3–5]. In this view, the body is not a device to simply be controlled by the brain, but rather is directly involved in making some behaviors less complicated for the nervous system.

In the domain of vocal production, vocal output is typically and reasonably thought to be controlled by a network of CPGs [6–11]; in some species, these CPGs may be activated, modulated, or suppressed by forebrain structures [12–14] (see [15] for a recent review). With regard to morphological computation, studies of birds [16–18], bats [19], and humans [20] reveal that the biomechanical properties of the larynx (or syrinx in birds) can simplify motor control of vocal production. For example, in zebra finches, discretely different song syllables can be produced by a simple linear driving force exploiting the soft tissue properties of the syrinx [16,17]. Along the same lines, simulating the biomechanical properties of the songbird vocal apparatus was also shown to reduce the number of control parameters needed by premotor neurons to organize song structure [12,21]. What is not known is the role that morphological computation may or may not play in vocal development.

Current investigations of the mechanisms of vocal development typically focus primarily on how changes at the neural circuit level lead to changes in vocal output. For example, the vocal learning literature emphasizes the role played by imitation and the neural changes that may facilitate this behavior, particularly in songbirds and humans [22]. In this case, vocal development is not restricted by body structure, but rather by memory- or motor-related constraints and perceptual predispositions. The possibility of morphological computation is not considered. However, in human infants (and, logically, all vertebrates), there is not only growth in the brain during vocal development, but also growth in the vocal apparatus (i.e., the larynx, the vocal tract, and the lungs) [23–25]. For example, in humans, lung volume nearly triples in size over the first 2 years [26]. Changes in these structures are likely to influence the development of vocal behavior in unexpected ways. Let us illustrate the point from a different domain of behavior: locomotion. A perfect example of morphological computation in development comes from a classic study of human infant stepping behavior [27]. Newborns are able to make well-coordinated stepping movements when held upright, but these movements disappear by the time they reach 2 months of age. While it was assumed by many that the change in stepping behavior was due solely to the developing nervous system (e.g.,[28]), Thelen and colleagues hypothesized that the loss of stepping behavior was due to body growth: the infants’ legs typically fatten up postnatally, and they do not yet have the strength to move heavier legs [27]. To test this hypothesis, they submerged the infants’ legs in water, effectively decreasing their mass. This resulted in the reappearance of stepping and thus falsified the alternative hypothesis that neural change was necessary [27]; the change in behavior was due to changes in the body.

**Fig 1:**
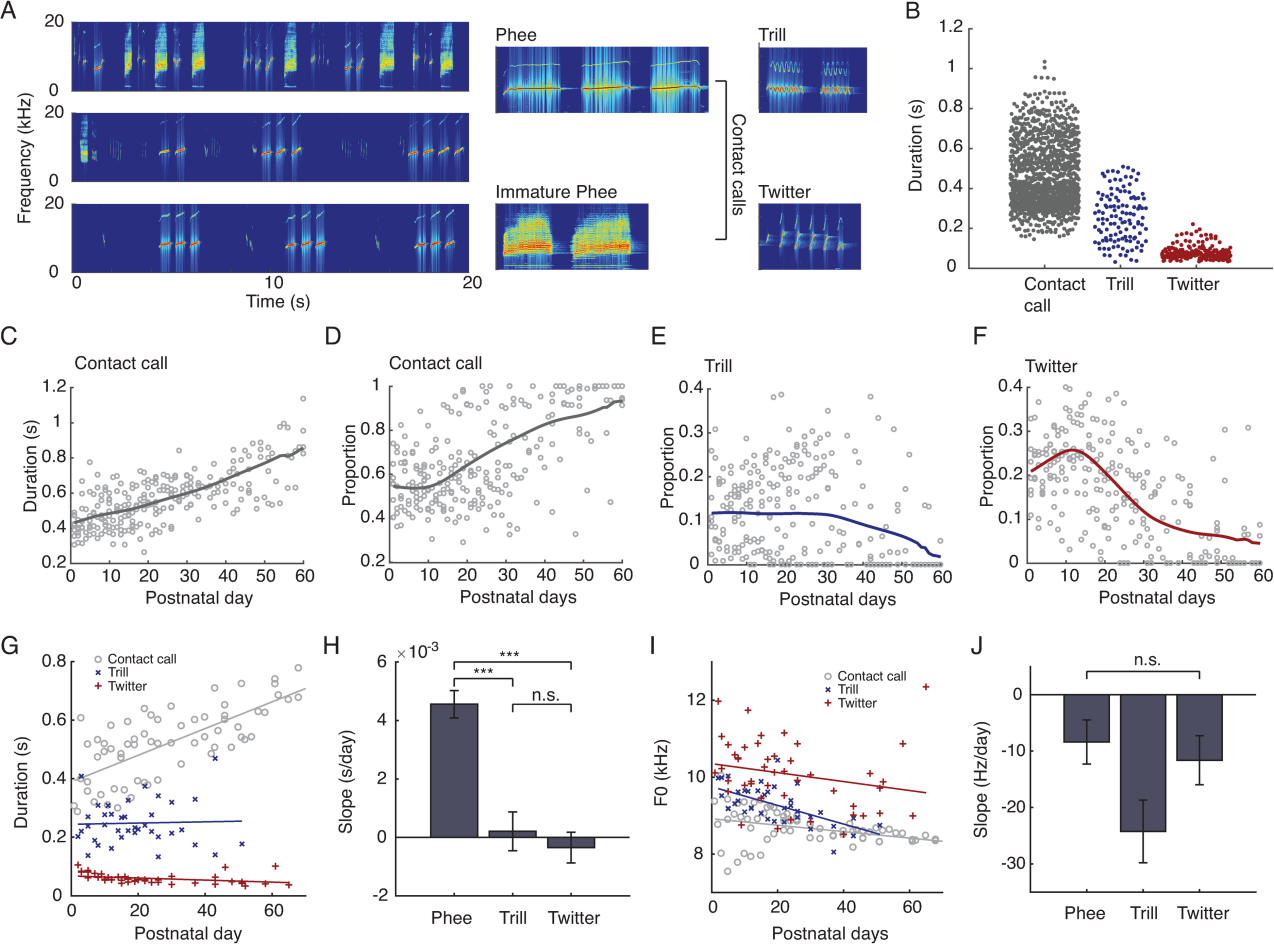
Developmental trajectories of marmoset vocalization. (A) Exemplars of marmoset infant babbling-like vocalization from different postnatal days and classification of distinct call types. (B) Comparison of the call duration of different call types from the first postnatal week (*n* = 1,208 contact call syllables, 121 trill syllables, 250 twitter syllables, F = 754.32, *p* = 1.52×10^−230^, ANOVA, each call type is different from other types). (C) Duration of contact calls over postnatal days (*n* = 13 subjects, 244 trials). (D–F) Proportions of contact calls, trill, and twitter over postnatal days (*n* = 13 subjects, 244 trials). (G) Duration and fundamental frequency (F0) change over time of three call types. Contact call increases in duration over time (*p* = 8×10^-10^), trill does not show significant change (*p* = 0.06), and twitter decreases over time in duration (*p* = 7×10^-4^). (H) Comparison of slopes for (G). The slope of the contact call change in duration is greater than trill and twitter (ANCOVA, F = 25.5, *p* = 3.6×10^-10^). (I) None of the calls show significant change in F0 over the first two months (*p* = 0.10, *p* = 0.09, *p* = 0.47 respectively for contact calls, trills and twitters). (J) Comparison of slopes for (I). There is no difference between the slopes of the F0 change (ANCOVA, F = 1.72, *p* = 0.18). Data underlying this figure can be found in S1 Data. F0, fundamental frequency.

We investigated whether developmental changes in vocal production are in part the result of morphological computation using marmoset monkeys as a model system. When out of visual contact of conspecifics (undirected context), adult marmoset monkeys exclusively produce and exchange contact “phee” calls [29,30]. Infant marmosets, however, produce mature and immature versions of this contact calls as well as calls that are inappropriate for the undirected context: trills and twitters [31,32]. Thus, in the undirected context, the goal of marmoset vocal development is to produce solely contact calls [31,32]. The infant trills, twitters, and contact calls have distinct spectral and temporal profiles (Fig 1A) [31,32]. Call duration, for example, readily distinguishes the syllables of trills, twitters, and contact calls (Fig 1B). The production of the longer duration contact calls is energetically costly (relative to trills and twitters), requiring sustained respiratory power [31,33,34]. Over the course of development, contact calls gradually increase in duration, becoming more adult-like; they increase in proportion as well [31] (Fig 1C and 1D). The short-duration trills and twitters, however, simply disappear over time in the undirected context (Fig 1E and 1F).

Given the energetics required to produce contact calls, we hypothesized that increases in lung capacity via body growth—without any developmental changes in the neural properties of the vocalization-related CPGs—can explain the disappearance of short-duration trill and twitter calls. As the lungs get bigger, the respiration rate slows down because inspiration and expiration take longer [35]; sensory feedback from the lungs to vocalization-related CPGs mediates this influence on vocal output [36]. Although there is also sensory feedback from the larynx [36], the vocal developmental data show much more pronounced changes in lung capacity–related duration of calls (Fig 1G and 1H) than in their laryngeal-related fundamental frequencies (Fig 1I and 1J). We believe this slowing of the respiration rate results in the disappearance of trills and twitters while increasing the proportion and duration of contact calls. To explore this possibility, we generated a numerical model of infant marmoset monkey vocal development and then tested model predictions by placing infant marmosets in a heliox environment and recording its effects on vocal production. The heliox environment simulates a developmental reversal of lung growth by increasing the rate of respiration. Our data show that the decreasing numbers of context-incorrect trills and twitter calls, and the increasing number and duration of the context-appropriate contact calls, are driven by morphological growth and not necessarily developmental changes in the intrinsic activity of neurons.

## Results

Let us provide the background on which our model is based. In previous studies, we showed that there is a slow 0.1 Hz oscillatory pattern in the spectral entropy and duration of vocalizations as marmoset infants produce highly stochastic vocal sequences [32,37]. In other words, there are alternating patterns of noisy (broad-band) and tonal calls, as well as long and short duration calls in these infant vocal sequences. These findings suggested that this complex vocal output is governed by neural dynamics that are under a strong influence of a low dimensional input that also exhibits such slow oscillations. We found that arousal levels—as measured by heart rate changes—accounted for this 0.1 Hz oscillatory pattern in vocal acoustics [32]. These findings translate into the following scenario: (1) when the animal is at its lowest or highest arousal levels, it produces immature (highest entropy) or mature (lowest entropy) sounding contact calls, respectively; (2) the shorter calls, twitters and trills, are generated between those two states. We thus sought a nonlinear model that can bridge the fluctuating arousal levels to the complex vocal behavior.

We built the model with the minimal set of control parameters that can generate infant marmoset monkey vocalizations. These consist of the subglottal pressure and the laryngeal tension [31,34]. This model provides the dynamics of the CPGs governing those two parameters. As the respiratory pressure energizes the vibration of vocal folds during the expiratory phase, it determines the duration of phonation. At certain pressure levels, laryngeal tension determines the fundamental frequency of the sound. In order to produce a long duration contact call, a long expiration is needed, as well as a constant laryngeal tension. In other words, the respiratory CPG has to oscillate at a slow rate while the laryngeal CPG is at a stable fixed point. To produce a trill call, respiration needs to provide a relatively sustained pressure while the laryngeal CPG provides a fast oscillating input. By contrast, a twitter call requires a strong, fast oscillation in respiration to break the sound into short syllables; this can be realized by coupling with the fast laryngeal oscillator.

**Fig 2:**
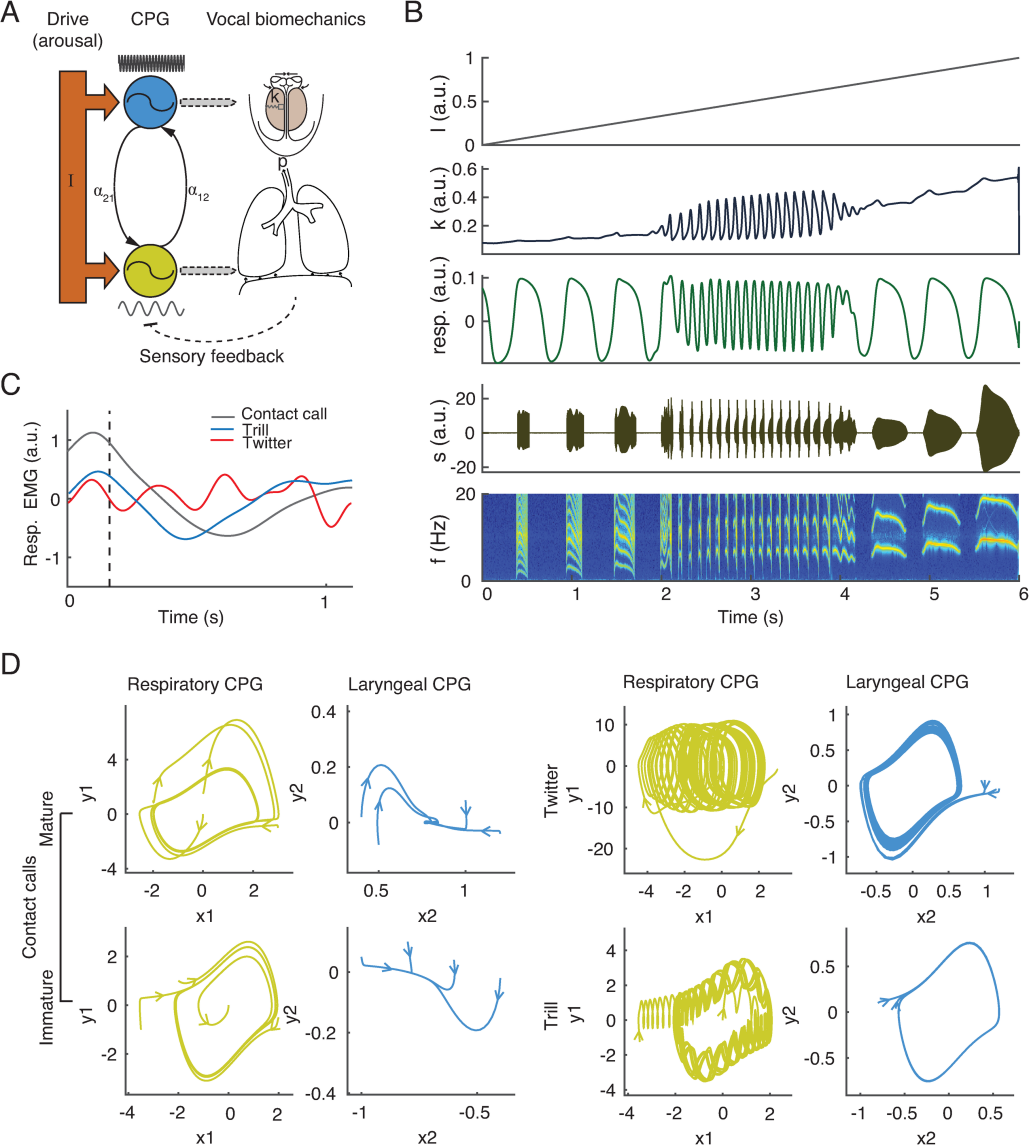
A central pattern generator model for infant vocal production. (A) Setup of CPG model. Both laryngeal (top) and respiratory (bottom) CPGs receive a common input (arousal). The CPGs coupled with each other. They drive the variation of the laryngeal tension and subglottal pressure to generate sound. Lung capacity affects the damping of the respiratory CPG via somatosensory feedback. (B) Different temporal patterns of the laryngeal tension and respiratory activity can be generated as the drive linearly ramps up, and distinct call types are produced. Panels from top to bottom: drive (arousal), laryngeal tension, respiratory activity, simulated sound pressure, and spectrogram. (C) Mean respiratory EMG profiles for the different call types. (D) Phase portraits in (x, y)-space illustrating the dynamics of the CPG model at different arousal levels. In regions of *I* (arousal) where the values are high or low, fixed points appear in the laryngeal dynamics, yielding mature and immature contact calls (left panels). In regions of moderate values of *I*, limit cycles appear in the laryngeal dynamics, which modulate the respiratory dynamics to produce trill or twitter (right panels). Within a panel, the left subplot is the phase portrait of the respiratory CPG and the right subplot is the laryngeal CPG. MATLAB code is available for figures (B) and (D) in S1 Code. CPG, central pattern generator; EMG, electromyography.

We thus set up our model with the topology that two distinct regions of stable fixed points are separated by an oscillatory regime (see Methods for details). The model is composed of two coupled oscillators with distinct natural frequencies: one CPG for respiration and the other for governing the oscillating tension of laryngeal muscles (Fig 2A) [38]. The activity levels of the CPGs are modeled by the amplitude of the oscillators. Both CPGs receive a common drive representing the arousal levels. This is similar to the linear drive used to generate nonlinear shifts in gait dynamics in spinal cord CPG models of locomotion [39]. In our autonomous model, the arousal input is the only time-dependent variable that drives the system across different dynamical regions. These CPG dynamics are then converted into air pressure and tension, which are fed into a biomechanical model of the marmoset monkey vocal apparatus [31,34].

One assumption key to the model is that the oscillations of the CPGs are adapted to the mass of the lungs [40,41]. It has been shown independently in a study of birdsong that the respiratory patterns can be generated in a Wilson–Cowan neural network integrated with the lung (air sac) dynamics via sensory feedback [42]. We also implemented a Wilson–Cowan model and found that the period of the oscillations is positively correlated with the mass of the organ (S1 Fig). We used this to justify setting up our main CPG model so that the CPG driving the lungs is much slower than the one driving the larynx. Furthermore, we implemented the effects of changing lung mass on the frequency of the oscillator by varying its time constant, i.e., greater lung mass yields slower oscillations.

In the initial model representing a postnatal day 1 marmoset infant (with a small lung size/damping coefficient), all marmoset call types can be generated by solely and linearly increasing the drive over the course of 6 s. Fig 2B shows that contact calls, trills, and twitters are generated (*s*, time-amplitude waveforms and *f*, spectrograms). This 6-s drive *I* is consistent with the ramping up phase of arousal fluctuations that underlie the production of real infant marmoset vocalizations [32]. The respiratory patterns generated by the model are qualitatively similar to the electromyography (EMG) recordings of marmoset infant respiratory patterns (Fig 2C) [32]. Low and high drive levels produce relatively constant CPG control of the laryngeal tension, *k*. This results in the spectrally flat contact calls (Fig 2D, left panels). Moderate drive causes the laryngeal CPG to oscillate around limit cycles, yielding trills and twitters (Fig 2D, right panels). The dynamics of the respiratory CPG are modulated by the laryngeal CPG via the coupling term. Depending on the oscillatory amplitude of the laryngeal CPG relative to the respiratory CPG, respiration can be shortened (trills) (Fig 2B; at 2 s and 4 s of respiration) or broken into minibreaths to produce twitters (minibreaths are the small respiratory oscillations added on top of a DC level [43]; Fig 2B; at approximately 2–4 s of respiration).

**Fig 3:**
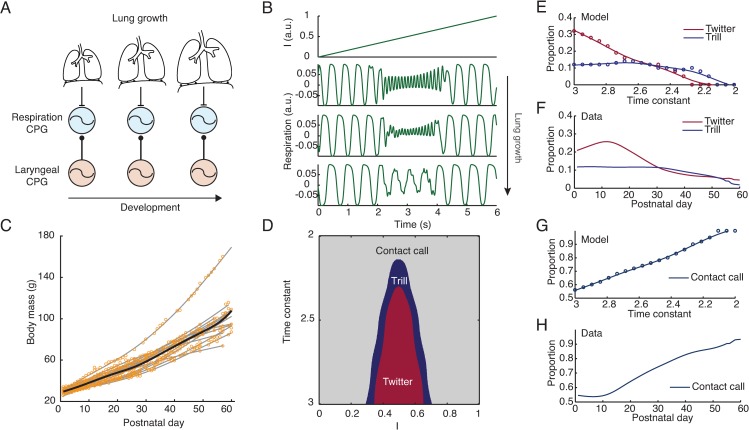
Simulated lung’s growth reproduces the developmental trajectories of call proportions. (A) Illustration of the impact of the lung’s growth on the respiratory CPG. (B) Simulated respiratory activity under ramping input with decreasing values of the time constant. Fast respiratory patterns diminish as time constant decreases. From top to bottom: γ_*1*_ = 3.4, 3.1, 2.8. (C) Body mass growth versus postnatal days (*n* = 13). Points are data, grey lines are cubic spline–fitted data, and black line is the mean body mass over time. (D) Diagram of different call types, classified by duration, in the parameter space of time constant and drive *I*. (E) Simulated twitter and trill proportions based on the areas in (D) at different time constants. (F) Averaged twitter and trill proportions from data (*n* = 13). (G) Simulated contact call proportions based on the area in (D). (H) Averaged contact call proportions from data (*n* = 13). MATLAB code is available for panels B, D, E, and G in S2 Code. CPG, central pattern generator.

We can now use the model to test the hypothesis that lung growth alone can account for the decreasing numbers of trills and twitters (Fig 1D and 1E) and increasing number and duration of contact calls (Fig 1C and 1F; see S1 Text for details). Fig 3A and 3B show that if we increase lung size, then we change the patterns of vocalization-related respiration. In this scenario, short duration respiratory patterns decrease in number (Fig 3B). Because the lung capacity scales proportionally with body mass [44] and body mass increases almost linearly with time over the first two months in marmosets (*n* = 13; Fig 3C), we fit the damping coefficient as a linear function of postnatal days to simulate a biologically plausible trajectory of lung growth. Fig 3D shows the proportion of call types as a function of the drive (*I*) level and increasing lung size. As the lungs grow, the range of *I* that generates trills and twitters decreases. The pattern of declining trills and twitters generated by the model is similar to the developmental pattern exhibited by real infant marmosets (Fig 3E and 3F), as is the increasing number of contact calls (Fig 3G and 3H). The best fit between the model and data was R^2^ = 0.77.

**Fig 4:**
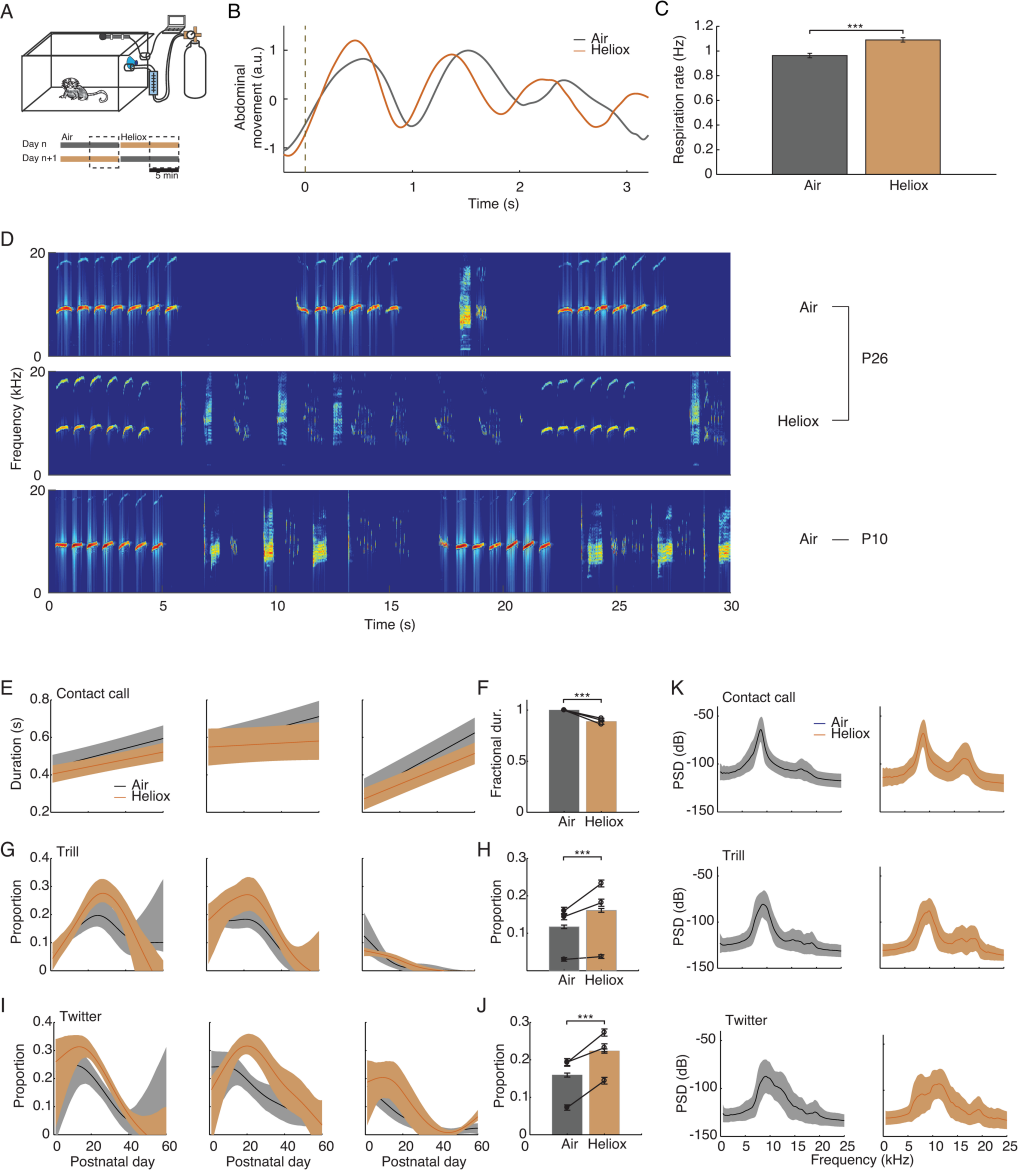
Heliox manipulation briefly reverses the developmental trend of vocal behavior. (A) Experiment setup. Infants (*n* = 2) were placed in the box for 20 min with 10 min for each condition (air versus heliox). The order of the conditions was counterbalanced across days. Only the last 5 min of each condition was used in the analyses. (B) Mean abdominal movements in air (*n* = 25 traces) and heliox condition (*n* = 37 traces) during the production of contact calls extracted from video. Data are from 2 subjects. (C) Respiratory rates in air (*n* = 25 video traces) and heliox (*n* = 37) condition during contact call production. Data are mean ± SEM. ***P < 0.001 (unpaired 2-tailed *t* test). (D) Vocal sequences produced in air and heliox on P26, in comparison with vocal sequences produced on P10 in air. (E) Duration changes over postnatal days for air and heliox conditions for each subject. Data are fitted to linear models. Shaded areas indicate 1 SE intervals. (F) Comparison of population mean of fractional contact call duration normalized to the air condition. Bar height represents population mean. Error bars are SEM. Each line is one subject. ***P < 0.001 (GLM). (G, I) Proportions of trills and twitters over postnatal days for air and heliox conditions. Shaded areas are the bootstrapped 95% confidence interval. (H, J) Comparison of heliox effect on trill and twitter proportion. Bar height represents mean proportion of all subjects (*n* = 3) and all trials over the first two months. Error bars are SEM. Each line is one subject ***P < 0.001 (GLM). (K) Heliox effect on sound spectrum. Left panels: mean power spectral densities (PSDs) of different call types in air condition. Right panels: mean PSDs in heliox condition. Data underlying this figure can be found in S1 Data. GLM, generalized linear model; heliox, helium–oxygen; PSD, power spectral density.

Our model shows that sensory feedback from growing lungs—without any changes to the CPGs themselves—can account for the decreasing proportion of trills and twitters. Thus, morphological computation is a plausible mechanism for vocal development in this case. To empirically test the model’s predictions, we manipulated the physical property of respiration by placing the developing marmoset infants in a helium-oxygen (heliox) environment. Because the air is lighter in a heliox environment, less time is needed to complete a respiratory cycle when the same amount of force is provided by the respiratory muscles (see S1 Text) [45]. An increase in the infant marmosets’ respiratory rate in the heliox environment would simulate the temporal dynamics of smaller lungs and allow us to test the predictions of lung growth on vocal output. We did this approximately every other day, from P1 to P60 (*n* = 3 subjects, *n* = 19, 19, and 27 sessions). For each session, we recorded vocalizations for 10 min in heliox and 10 min in air. The order of these two conditions was counterbalanced. To allow for gas concentration to stabilize when transitioning between heliox and air, we only analyzed the vocalizations in the last 5 min of each 10-min interval. For two infant marmosets, we measured the heliox-induced change in respiration rate via video analysis of abdominal movements while they produced contact calls simply to confirm the obvious: that respiration rate should increase in the heliox. Fig 4B shows averaged traces of respiration of two infants in heliox (*n* = 37 traces) and in air (*n* = 25 traces), demonstrating that there is an increase in the rate of respiration during the production of calls. Fig 4C shows the mean increase in the respiration rate across the two infants (24.9% ± 3.6% increase, mean ± SEM; *p* = 4.0×10^−8^, unpaired 2-tailed *t* test).

As predicted by the model, placing infant marmosets in heliox increased the proportion of trills and twitters and decreased the duration of contact calls, a reversal of the vocal development trend (Fig 4D). Fig 4E, 4G and 4I show the developmental trajectories of these three call types produced in and out of the heliox environment for all three infants. For all of them, more trills and twitters are produced, and the duration of contact calls shortened, in heliox. Fig 4F, 4H and 4J show the mean change between heliox and air over the first two months. The proportion of trills increased significantly in heliox (36.5% ± 6.0% increase, mean ± SEM; *p* = 1.45×10^−9^, effect size = 0.134, generalized linear model [GLM]), as did the proportion of twitters (41.8% ± 5.3% increase, mean ± SEM; *p* = 1.80×10^−15^, effect size = 0.266, GLM). Contact call duration under heliox condition dropped by 11.0% ± 0.2% compared to those produced in air (mean ± SEM; *p* = 0, effect size f2 = 0.13 with power = 1.0, GLM). To rule out the possibility that the heliox manipulation affected the animal’s arousal levels and thus consequently caused the observed differences in vocal output, we performed pairwise comparison on the call rate (number of calls produced per min) between these two conditions. Increased call rates are typically associated with increased arousal levels [46]. We did not observe differences in the amount of calls produced as function of heliox versus air (*p* = 0.65, Wilcoxon signed rank test). These data demonstrate that developmental changes in lung capacity can account for the changing call types produced as the infant marmoset grows.

An additional possibility is that the heliox makes it easier to produce trills and twitters via a laryngeal influence, as it is well known that heliox can affect the spectral properties of vocalizations [47–49]. When compared to vocalizations produced in air, heliox shifts the resonant frequency of the vocal tract (the oral and nasal cavities) [50], enhancing the second harmonic of the vocalizations’ spectra (S1 Text). However, heliox does not have a large effect on the fundamental frequency (F0) [47–49], and the F0 represents the source sound coming directly from the larynx [50]. We calculated the mean power spectral density (PSD) of contact calls, trills, and twitters across all postnatal days in both heliox and air. All three call types had nearly identical F0s (Fig 4K, left panels). Heliox significantly enhanced the second to first harmonic amplitude ratio of all tonal calls by approximately 18 dB (*p* = 1.0×10^−63^, effect size d = 0.23, unpaired 2-tailed *t* test). In contrast, the F0s of the tonal calls were increased by only approximately 1.8% in heliox (*p* = 5.4×10^−27^, unpaired 2-tailed *t* test, however with a very small effect size d = 0.14). Thus, the heliox effect on the spectral properties of vocalizations was mostly passive and only minimally due to changes in the effort for laryngeal control. That the heliox environment had largely the same effect on all three call types suggests that air density does not differentially benefit the production of trills and twitters.

## Discussion

Vocal development is a consequence of many interacting factors, including the growth of the vocal apparatus, the muscles that innervate it, the nervous system that controls those muscles, and social interactions that adjust nervous system function via experience [34,51]. Previous efforts isolated the role of social interactions on marmoset monkey vocal development [31,52,53]. Those studies took advantage of individual differences in the amount of social feedback provided by parents while controlling for the contributions of body growth. They found that the rate of developmental changes in some acoustic parameters, such as the noisiness and amplitude modulation, could be attributed to the amount of social feedback provided by parents, while other parameters, such as duration, dominant frequency, and the disappearance of calls produced in the incorrect context, could not be explained with such experience-dependent mechanisms [31,52]. Conversely, in the current work, we found that increases in call duration and changes in call usage (i.e., the disappearance of calls produced in the incorrect context) could be attributed solely to the growth of one part of the vocal apparatus—the lungs, which provide the respiratory power to produce vocalizations.

Our model of interconnected laryngeal and respiratory CPGs predicted that if the respiratory CPG received sensory feedback from growing lungs, the production of trills and twitters would decrease and the production and duration of contact calls would increase. No changes in the neural properties of the CPGs were required. These predictions were empirically tested by recording the vocalizations of infant marmosets in a heliox environment. Akin to placing infants in water to reduce the load on stepping behavior [27], placing vocalizing infant marmoset monkeys in heliox reduces the respiratory load, thereby increasing the number of trills and twitter calls and shortening the contact calls. Thus, in contrast to the strong emphasis on neural changes typically used to explain vocal development [22], these data support the idea that some aspects of vocal development can occur through morphological computation: The body (in this case, the growing lungs) can be exploited as a computational resource by reducing the number of control parameters that need to be tracked and adjusted by the nervous system [3–5].

Respiration plays a key role in both vocal production [8,54] and behavior in general [55]. Respiration and locomotion, for instance, are synchronized to different extents depending on the mechanical constraints imposed by posture and body size on respiration [56]. The upright posture of humans reduces the influence of gait on respiration, allowing more flexibility in respiratory patterning [56]. Our model proposed that morphological computation through lung growth benefited the neural control of vocalization by weakening the coupling of respiration from laryngeal movements. The apparent decoupling of respiration from laryngeal influence by lung growth in marmosets may in the same way allow more independent control of respiration, thereby improving the accuracy of vocal communication. Naturally, our model is a simplification of what is known about the dense, multinode network of vocalization-related CPGs [10], which includes within it a complicated network of respiratory CPGs [57]. Nevertheless, our model and behavioral data provide supportive evidence for the hypothesis that the intrinsic properties and connectivity of these networks need not change over the course of vocal development to account for some dramatic shifts in vocal output.

It is important to note that the model presented in this work provides only one possible solution to the structure of the neural activity that can generate sequences of marmoset infant vocalizations. We did not design the model to simulate any specific neuroanatomical or neurophysiological details, as these are not yet well understood. Rather, we use the model as a way to extract a low-dimensional representation of this complex vocal behavior. There are other dynamical models with similar structures that can also lead to the same results. For example, an alternative setup of the CPG model would be one with articulate CPGs driving different neural populations that, respectively, drive the laryngeal and respiratory muscles. Although our study suggested that the bifurcations that create different vocal patterns occur at the level of subcortical CPGs, it is not sufficient to refute the alternative possibility that forebrain structures might also play an important role [14,58,59].

Given that vocal development consists of a number of “moving parts” in the body and the brain, we need to understand how these parts and their relationships change over time to produce mature vocal behavior [34]. This integrative understanding is important from a clinical perspective as well. Human infants who do not vocalize a lot tend to be fed and held less by mothers, and are slowed in their speech development [60]. The lack of adequate early vocal output by infants may be due to many factors, including problems related to nervous system function such as arousal dysregulation or motor control deficits, weak laryngeal and respiratory muscles, and/or abnormal growth of the vocal apparatus: the larynx, orofacial cavity, and lungs. It is important that one considers the “whole system” when trying to understand how any behavior works or may go awry.

## Methods

### Ethics statement

All experiments complied with the Public Health Service Policy on Humane Care and Use of Laboratory Animals and were approved by the Princeton University Institute Animal Care and Use Committee (protocol number 1908–15).

### Subjects

The vocal development trajectory was constructed partially from a subset of previously published dataset (*n* = 10 subjects) [31] and partially from the control condition of the three subjects used in this work. The subjects are infant common marmosets (*Callithrix jacchus*) housed at Princeton University. The colony room is maintained at approximately 27°C and 50%–60% relative humidity, with 12L:12D light cycle. The subjects were all born in captivity and raised by family. All subjects, including all other members in the family, received water ad libitum and were fed with standard commercial chow supplemented with fruits and vegetables. All experiments were approved by the Princeton University Institute Animal Care and Use Committee.

### CPG modeling

In the search for an appropriate model, we considered a two-dimensional system for each CPG oscillator to allow Hopf bifurcations. We also looked for a model that contains two regions of stable fixed points separated by a limit cycle region via Hopf bifurcations. For simplicity, we start building the dynamical model for each oscillator from a simple 2D system

$$\left\{ \begin{array}{c} \dot{x}=y\\ \dot{y}=f(x,y,a) \end{array}\right.,$$

in which *f*(*x*,*y*,*a*) is a polynomial up to the third order and *a* is a parameter. To allow the location of the fixed point to change from low to high values as the parameter varies monotonically, we simply let x* = *a* be the only fixed-point solution in our model. Thus, *f*(*x*,*y*,*a*) can have the form *f*(*x*,*y*,*a*) = *σ*(*x*−*a*) + *g*(*x*,*y*)*y*, where *σ* is a constant and *g*(*x*,*y*) is a polynomial up to the second order. With these simplifications, the Jacobian matrix has the form

$J=\left(\begin{array}{cc} 0 & 1\\ \sigma & g(a,0) \end{array}\right)$. To have Hopf bifurcations occurring twice, *σ* < 0 and *g*(*a*, 0) switches signs twice; thus, it can be a parabola passing 0 twice. In addition, to allow oscillations in the middle range of *a*, the parabola is inverted. Hence, *g*(*x*,*y*) can have the form *g*(*x*,*y*) = *μ*(*b* – *x*^2^) + *h*(*y*), where μ < 0 and b > 0 are constants and *h*(*y*) is a polynomial with the lowest order of one and highest order of two. Again, we drop *h*(*y*) for simplicity. Without losing generality, we let σ = −1, μ = −1, and b = 1. We also introduce a coupling term from the other oscillator in the equation and a time constant to change the oscillating frequency. The complete model is as follows

$$\left\{ \begin{array}{c} {\dot{x}}_{i}=y_{i}\\ {\dot{y}}_{i}=\gamma_{i}^{2}a_{i}-\gamma_{i}^{2}x_{i}+\gamma_{i}y_{i}-\gamma_{i}x_{i}^{2}y_{i}+\gamma_{i}^{2}\kappa (x_{j},y_{j}) \end{array}\right.,$$

in which *a*_*i*_ is the drive input to oscillator *i*, *γ*_*i*_ is the time constant for oscillator *i*, and *κ(x*_*j*_,*y*_*j*_) is the coupling input from oscillator *j*. We assume that the coupling is linear and let *κ* = *α*_*ji*_*y*_*j*_, in which *α*_*ji*_ is the coupling strength. Greater *γ*_*i*_ corresponds to faster oscillation. We define $I=\frac{a-a_{min}}{a_{max}-a_{min}}\in [0,1]$ for the relative drive strength.

The parameters of the model are listed in Table 1. The sensitivity of the model’s dependence on the parameters is analyzed in S3 Fig.

**Table 1:** 

Parameter	Value
*γ*_1_	2–3
*γ*_2_	25
*α*_21_	4
*α*_12_	0.015
*a*_1_	[−0.1, 0.1]
*a*_2_	[−1, 1]
*p*_0_	0.1
*k*_0_	32
$x_{2}^{0}$	5

We saturated *x*_1_ and *x*_2_ with sigmoid functions to get biologically reasonable *p* (air pressure, varying between–*p*_0_ and *p*_0_) and *k* (laryngeal tension, varying between 0 and *k*_0_):

$$\left\{ \begin{array}{c} p(t)=p_{0}\tanh\left(x_{1}\right),\\ k(t)=\frac{k_{0}}{1+e^{-(x_{2}-x_{2}^{0})}}. \end{array}\right.$$

The behavior of the CPG dynamics was visualized using the phase portraits, in which the oscillatory amplitude *x*_*i*_ was plotted against the velocity *y*_*i*_ = *dx*_*i*_/*dt*. With different values of the input, different dynamics were produced.

### Call proportion simulation

We estimated the proportion of different call types using the bifurcation diagram of *x*_1_ in the parameter space of I and *γ*_1_. As different call types are characterized by different duration (Fig 1B), we used the spectrum of *x*_1_ to find the regimes for different calls. For each combination of *I* and *γ*_1_, we iterated in solving the ODE using the Runge–Kutta method 2,000 times (after we discarded the first 500 iterations) with 0.01 step size. The regions for different call types were identified based on the oscillatory frequencies. Call proportions were estimated as the range for call type i in the [0, 1] range.

To compare the model with real data, we found the parameters *β*_0_ and *β*_1_ for the linear transform *PND* = *β*_0_ + *β*_1_*γ*_1_ that led to the least sum of squares $\beta_{0},\beta_{1}=\underset{\beta_{0},\beta_{1}}{\mathrm{argmin}}\sum_{i=1}^{2}\sum_{j=1}^{N}{\left(p_{i}(\gamma_{1}^{j})-{\tilde{p}}_{i}(\beta_{0}+\beta_{1}\gamma_{1}^{j})\right)}^{2}$, where *p*_*i*_ and ${\tilde{p}}_{i}$ are the simulated and real proportion of call type i (twitter and trill). To estimate the goodness of fit, we calculated the R^2^ between data and simulated proportions.

### Heliox experiment

Starting from P1, marmoset infants were placed in an induction chamber that holds approximately 45 L of air. The subjects were introduced into the chamber through the lid on top of the chamber. Heliox (20% oxygen and 80% helium) was passed through the inlet on the chamber and air was expelled from the outlet (Fig 3A). An air flow meter was attached to the inlet. A microphone (Sennheiser MKH 416-P48) was placed inside the chamber to record vocalizations. To reduce echoes, acoustic foam was attached to the walls of the chamber. An oxygen sensor (PASPORT Oxygen Gas Sensor-PS-2126A) was placed inside the chamber to monitor oxygen concentration throughout the experiment. In the control condition, we replaced the solid lid with a perforated lid. In each session, we carried out recordings of 10 min in heliox and 10 min in air. The order of these two conditions alternated every session. Since it requires 5 min for the gas to fill up the chamber, we discarded the first 5-min recording in both heliox and air conditions in the analysis. Heliox was provided constantly through the heliox session. To control the auditory effect from the heliox injection, we recorded the sound of airflow in the chamber and played it through a Bluetooth speaker (Lyrix Jive Jumbo) placed in the chamber near the inlet during the control condition. The sound pressure level of the playback was calibrated the same as the actual airflow sound using a sound level meter (Extech 407730). An HD webcam (Logitech C930e) was placed in front of the chamber facing the side where there was no foam attached to record the abdominal movement during vocalization at 30 fps.

### Respiratory activity extraction

To test if the heliox approach was effective, we extracted respiratory pattern of the abdominal movement duration vocalization from video recordings (Logitech C930e). Phonation requires about 5- to 30-fold of pressure more than the baseline breathing, and therefore, it depends upon the abdominal sheet to drive active expiration during vocalization [61,62]. We extracted abdominal movements from video recordings in two marmosets who were approximately 2 months old during the production of phee calls in air and heliox environments. Infants at this age essentially only produce phee calls in isolation. Movie clips during phee call production were segmented using Windows Movie Maker. The marmosets usually stay still during vocalization, and so we could select a rectangular area around the abdomen through the frame stack and track its movements during the time window of vocalization. The RGB images were converted to grayscale by taking the mean across the color dimensions. The areas were first vectorized and converted into a matrix with rows representing frames and columns representing pixels. Principle component analysis was carried out to capture frame-to-frame variations related to respiration. The principle components were then aligned with the sound signal, and the PCs that were correlated with vocal production were selected to represent the abdominal movements during vocalization (S2A Fig). The average traces of the video extractions were calculated from the resampled data at 100 Hz and were aligned to the call onsets. We averaged the traces of the same condition. To compare the respiratory rates in different conditions, we calculated number of cycles per s using number of cycles divided by total call duration. To justify this method, we also compared the results with EMG recording (S2B Fig).

### Data processing

Onsets and offsets of individual utterances were automatically detected using a custom-made MATLAB routine. Call types were first categorized automatically based on duration and Wiener entropy and then manually inspected. Duration was calculated as the duration of individual utterances within a call. Consecutive utterances in the same category with no more than 0.5-s gaps were grouped as one call. Each point of the call type proportions was calculated by grouping two consecutive, counterbalanced sessions. Call proportions were calculated as the number of calls of a specific type divided by the total number of calls in this condition. The corresponding postnatal days were calculated as the mean of the two consecutive days.

The PSD of the vocalizations (per syllable) was estimated using Welch’s method by applying the MATLAB *pwelch* function. The F0 was identified as the first peak of the sound spectrum. The second harmonic (F1) was identified as the second peak of the spectrum. The amplitude ratio between F1 and F0 was calculated as the ratio of the mean amplitudes at F1 and F0 within a syllable.

### Statistical analysis

We used MATLAB *csaps* function to fit the data over the first 60 postnatal days for individuals. The 95% confidence intervals were constructed by randomly sampling the data with replacement 1,000 times and fitting cubic spline using *csaps* for each bootstrap sample. MATLAB *fitglm* routine was used to fit the GLM to the occurrences of trill or twitter over the first two months in all three subjects. In this model, we tested the effect of heliox condition and also controlled for individual differences. We assumed that the response variable follows binomial distribution, and in Fig 4G, we fitted a multiple logistic regression model for the occurrences of trill

$$logit(I_{trill})=\alpha +\beta_{1}*S_{2}+\beta_{2}*S_{3}+\beta_{3}*I_{heliox}+\epsilon ,$$

where *S*_2_ and *S*_3_ are dummy variables for subject #2 and #3 encoded as *S*_1_ = 00, *S*_2_ = 01 and *S*_3_ = 10, *I*_*heliox*_ = 0 or 1 for air condition and heliox condition and *ϵ* as the random error.

Similarly, in Fig 4I, we fitted the model

$${logit(I}_{twitter})=\alpha +\beta_{1}*S_{2}+\beta_{2}*S_{3}+\beta_{3}*I_{heliox}+\epsilon .$$

We used the fitted β_3_ and its standard error to estimate the mean difference in proportion between the two conditions with subject difference taken into account. The significance of the heliox effect was accessed by the *p*-value of *β*_3_ from the *fitglm* output. To estimate the effect size of the GLM, we compared the areas under the receiver operating characteristic (ROC) curves for a model with the condition variable included and one without it [63]. The area under the curve (AUC) was calculated using the MATLAB routine *perfcurve*, and the ratio of AUC was calculated as

$$r=\frac{AUC_{c}-AUC_{0}}{AUC_{0}-0.5},$$

where *AUC*_*c*_ is the AUC with the condition variable and *AUC*_0_ is the one without that variable. As in practice we compared the area above the diagonal line, we subtracted 0.5 in the denominator.

To evaluate the heliox effect on duration, we calculated the duration of the contact call syllables under each condition as a fraction of the daily mean duration in air condition. We assumed that the fractional duration is normally distributed and fitted a general linear model to the fractional duration as a function of subject identity #2 and #3 and condition

$$d=\alpha +\beta_{1}*S_{2}+\beta_{2}*S_{3}+\beta_{3}*I_{heliox}+\epsilon .$$

*β*_3_ was then used to estimate the reduction of syllable duration. The effect size was estimated using Cohen’s f^2^ method for multiple regressions. Power analysis was carried out using the G*Power 3.

The spectral features, F0 and amplitude ratio of F1/F0, were compared between the two conditions using unpaired 2-tailed *t* test. Effect size d estimation and power analysis were performed in G*Power 3.

## Supporting information

S1 Fig Change of neural dynamics due to lung growth via feedback. (A) Schematic of the integrated neural-mechanical respiratory system with sensory feedback. An excitatory-inhibitory neural network drives the motor neurons in the spinal cord that subsequently drives lung movement. The lung volume also provides negative feedback to the neural network. (B–D) Oscillations of the lung volume, inhibitory neuron and excitatory neuron at different values of lung mass. Heavier lungs produce slower oscillations. Model parameters: *E*_1_ = −2, *E*_2_ = −2, *τ* = 0.31, *k* = 5 and *μ* = 1.
(EPS)

S2 Fig Video extraction of respiratory activity. (A) An area of the abdomen with clear movements during vocalization was extracted from the video. The frames were vectorized and converted to an *m*-by-*n* matrix with *m* the number of pixels and *n* the number of frames. PCA was performed on this matrix and a representative principle component was used for quantifying abdominal movement. (B) Comparison between video extraction of abdominal movement and EMG recording of the respiratory activity.
(EPS)

S3 Fig Sensitivity test. (A–B) Proportion simulation at different parameter values. The curves are shifted at different parameter values but the result is qualitatively similar.
(EPS)

S1 Text Supplemental text. (DOCX)

S1 Data Data underlying Figs 1 and 4. (XLSX)

S1 Code MATLAB code for Fig 2B and 2D. (M)

S2 Code MATLAB code for Fig 3B, 3D, 3E and 3G. (M)

## Abbreviations

AUC: area under the curve
CPG: central pattern generator
EMG: electromyography
F0: fundamental frequency
GLM: generalized linear model
heliox: helium–oxygen
PSD: power spectral density
ROC: receiver operating characteristic
