# Supplementary material for: Vocal development through morphological computation
Source: PLoS Biol. 2018 Feb 20;16(2):e2003933. doi: 10.1371/journal.pbio.2003933 (PMC5834215; doi:10.1371/journal.pbio.2003933)
Supplement: S1 Text — (DOCX) [file pbio.2003933.s004.docx]

**Supplemental Text**

*A feedback model for a neural-mechanical system*

The respiratory pattern can be generated via a neural-mechanical model [1]. We simulate the lungs of the marmoset as a mass-spring system with damping. The variable *x* is the lung expansion measured from equilibrium at atmospheric pressure. The muscle that drives respiration is activated by neural input from the motor neurons. For simplicity, the motor neuron in spinal cord sums the input from brainstem with some weights. We use a Wilson-Cowan model to describe the activity of the excitatory and inhibitory network in the brainstem. The neurons also receive sensory feedback of the lung pressure. The complete system is modeled by the following equations:

$$\left\{ \begin{aligned} m\ddot{x}=-kx-\mu\dot{x}+10(2v_{E}-v_{I}) \\ \tau\dot{v}_{I}=-v_{I}+f(I_{1}+v_{E}) \\ \tau\dot{v}_{E}=-v_{E}+f(I_{2}-v_{I}+4v_{E}-g(x)) \end{aligned}, \right.$$

where $f\left( x \right)=1/(1+e^{-x})$ and $g\left( x \right)=9x^{3}/(1+x^{3})$ (adapted from [1]).

To simulate lung growth, we increased the mass *m*. The oscillatory frequency of the lungs in this system is inversely correlated with the lung mass (S1 Fig). In this simple implementation, we demonstrate the plausibility that the rhythmicity of the neural activity can adapt to the change of body size. We also aim to use this model to justify the idea of using the change of the time constant to simulate lung growth.

*Biomechanical modeling*

We adopted a previously described model of the marmoset vocal apparatus to simulate the dynamics of vocal production and computationally reproduce vocalizations. This model approximates the lateral movement of larynx as a one-mass oscillator with nonlinear damping. The oscillator is driven by the pushing force of the air pressure. While the complete set of equations has been described in [2], we emphasize here the key equation governing the larynx position as following:

$$\ddot{x}+p(t)\gamma^{2}+k(t)\gamma^{2}x+\gamma^{2}x^{3}+\gamma x^{2}\dot{x}-\gamma^{2}x^{2}+\gamma x\dot{x}=0.$$

The subglottal pressure is denoted $p$ and the laryngeal tension $k$. $\gamma$ is introduced for parameter fitting. The glottal flow is fed into a vocal tract, modeled as a uniform cylindrical tube, generating a filtered sound with frequencies around resonance enhanced (see [2] for details). The pressure at the vocal tract entrance is calculated from $x$ as following: $P_{in}(t)=c_{1}x(t)+c_{2}\dot{x}(t)-c_{3}\ddot{x}(t)-rP_{in}(t-T_{s}),$
where $c_{1}$, $c_{2}$, $c_{3}$ are coefficients for Taylor expansion terms of the function relating $x$ and $P_{in}$, $r$ is reflection coefficient representing the portion of pressure bounced back from the mouth, $T_{s}$ is the sound travel time through the upper vocal tract to the mouth. Eventually, the uttered sound pressure is given by

$$P_{sound}(t)=(1-r)P_{in}(t-T_{s}/2).$$

Heliox mainly changed the resonance frequency of the vocal tract filter by shortening the sound travel time $T_{s}$, but it minimally altered the dynamics of the vocal fold.

*Heliox effect on respiratory rate*

If we assume that pressure exerted to the lungs ($P_{lung}$) by the respiratory muscles in these two environments to achieve the same subglottal pressure ($P_{s}$) is the same and that total volume of gas exhaled is the same, according to Bernoulli’s principle, we have 
$P_{lung}\approx P_{s}+\frac{1}{2}\rho_{H}v_{H}^{2}=P_{s}+\frac{1}{2}\rho_{A}v_{A}^{2}$,
where $\rho_{H}$ and $\rho_{A}$ are densities of heliox (0.5 g/L) and air (1.25 g/L), $v_{H}$ and $v_{A}$ are airflow speeds of heliox and air. For steady expiration (airflow speed is constant), we have the relationship between gas volume in the lungs and expiratory duration $V_{lung}=T_{H}Av_{H}=T_{A}Av_{A}$, in which $T_{H}$ and $T_{A}$ are expiratory duration in heliox and in air respectively, $V_{lung}$ is the volume of lungs and $A$ is the sectional area of the glottis. Under the above assumptions, the theoretical ratio of respiratory rate in heliox to that in air is $\frac{R_{H}}{R_{A}}=\frac{T_{A}}{T_{H}}\approx\sqrt{\frac{\rho_{A}}{\rho_{H}}}\approx1.67$. The real effect on respiration was smaller than this theoretical value, possibly due to the less total work of respiration.

*Heliox effect on vocal tract resonant frequency*

For sound waves traveling in ideal gas within a closed-end tube, one can derive the particle displacement at the outlet of the tube as $S\left( L,t \right)=\frac{x_{0}}{coskL}e^{-i2\pi ft}$, in which $k=2\pi f\sqrt{\frac{\rho}{\gamma p_{0}}}$, where *f* is the sound frequency, $\rho$ is the mass density of the gas, $\gamma$ is the heat capacity ratio of the gas and $p_{0}$ is the equilibrium pressure, $x_{0}$ is the amplitude of the source oscillation and $L$ is the vocal tract length. The vocal tract gain thus is $G=\frac{1}{coskL}$. The first resonance occurs when the length of the tube is a quarter of the sound wave length with the resonant frequency $f=\frac{1}{4L}\sqrt{\frac{\gamma p_{0}}{\rho}}$. Thus the ratio of resonant frequencies in heliox vs. in air is $\sqrt{\frac{\gamma_{a}\rho_{h}}{\gamma_{h}\rho_{a}}}\approx1.85$, where the subscripts *a* and *h* denote air and heliox. By plugging in the atmospheric pressure for $p_{0}$, letting $\gamma=1.40$ and $\rho\approx1.205$ kg/m^3^ and using 9 mm for the approximated vocal fold length [3,4], the resonant frequency in air condition is around 9.5 kHz, close to the F0 of the tonal calls. In heliox condition, using $\gamma=1.59$ and $\rho\approx0.399$ kg/m^3^ yields the resonant frequency at around 17.6 kHz, which means that the second harmonic of the tonal calls should be amplified in heliox.

*Comparison between video extraction and EMG recording*

To justify the video extraction method, we compared the principle components of the abdominal movements with the EMG recordings at the abdominal muscles around the lower ribs in an adult marmoset. The EMG signals was taken from three surface electrodes (Genuine Grass surface electrode) that were sewed on an elastic band. Signals from the electrodes were deferentially amplified using instrumental amplifier (AD620) and acquired at a sample rate of 1 kHz by the Plexon MAP system (Plexon). In order to estimate how well the EMG signals matched the video extraction, we resampled both signals to 100 Hz and calculated the maximal value of their cross correlation (S2B Fig). The maximal cross correlation between these two signals is 0.57±0.03 (mean±SEM), significantly greater than chance (with upper limit of the 95% confidence interval of 0.18). Thus, video extraction performs similarly to the EMG recording.

**References:**

1. Trevisan MA, Mindlin GB, Goller F (2006) Nonlinear model predicts diverse respiratory patterns of birdsong. Phys Rev Lett 96: 058103.

2. Takahashi DY, Fenley AR, Teramoto Y, Narayanan DZ, Borjon JI, et al. (2015) The developmental dynamics of marmoset monkey vocal production. Science 349: 734-738.

3. Schmidt MF, Goller F (2016) Breathtaking Songs: Coordinating the Neural Circuits for Breathing and Singing. Physiology 31: 442-451.

4. Teramoto Y, Takahashi D, Holmes P, Ghazanfar AA (2017) Vocal development in a Waddington landscape. eLife 6: e20782.
